# An Inconsistent Iconoclast

**Published:** 1906-12

**Authors:** C. P. Davis

**Affiliations:** Americus, Ga.


### AN INCONSISTENT ICONOCLAST.

By C. P. Davis, D. D. S., Americus, Ga.

Georgia State Dental Society 1906.

You know that Webster defines an "Iconoclast" as "an image breaker," or literally "one who would change the established customs, or general way of doing things."

Now, today let us point out to you just one established custom in our profession and all try to be Iconclasts.

We can never reach perfection here, but if we have our standards *ideal,* our *real* accomplishments will surely improve.

The particular thing I wish to call your attention to and protest against is a practice common to all, or nearly all of us. I am guilty myself, hence the title of this paper; that is the use of *different metal* in the *same mouth.* Every day we hear from our patients, "Doctor, put gold in my front teeth and silver in those that won't show." You've heard it, haven't you? And if you are like your Inconsistent Iconoclast you do that very thing.

Generally it's done because the average patient hasn't enough money to pay for all gold, and you and I have to live by our professional income, and just cannot use thirty dollars worth of time for a ten dollar fee. The fellow who practices dentistry for love of it and thinks he can benefit the greatest number of people by dental operations, can do that, maybe, though I don't know him.

It is a fact, and I have no fear of being contradicted when I reiterate it, that time and time again, though we do use gold in front teeth and amalgam in the posterior teeth even when our patients are willing and able to pay for it. Why?

I won't tell you lazy man that it's because you are just too lazy to learn to work gold in bicuspids and molars, for you know that already, yet you won't exert yourself and learn.

Neither will I tell you bungling stone masons that it requires much more skill to insert gold fillings or inlays in cuspids, bicuspids or molars than it does amalgam, because you know that already.

Neither will I tell the "cheap" man that it's because it requires so much energy, skill and time to use gold than it does amalgam that he cannot put gold in posterior cavities as his advertised or know fixed "price per filling," that he couldn't do it if he would.

But, really, though, it's because it's all of these as well as a tax on our patients' endurance as well as purse that we don't, but to the majority of us it's on account of ignorance on our part. Just a few men, comparatively, in the profession have found out by clinical experience that where there are elaborate gold fillings or gold crowns or bridges in a mouth that amalgram fillings would not stay "put," and hence have abandoned amalgam almost entirely for gold, saying amalgam is not fit to fill a tooth with. They did not know *why* that amalgam would not stay, but I do.

I am not today advocating the use of gold only, for I believe in amalgam as well as gold, for I have seen good amalgam filling forty years old, looked good for forty more, but there was no gold in that mouth, and what I wish to impress upon you is to discontinue the use of two or more metals in the same mouth. Naturally you ask what reason I have for this advise, and once again I say "Electrolysis."

With gold and amalgam in the same mouth whenever the saliva in that mouth becomes acid you at once have electrical action and your amalgam is electrically dissolved and deposited upon your gold, silver plating and turning it dark, at the same time leaving microscopic fissures between your amalgam and tooth, and you know that means recurrent decay.

F. J. Brislee, M. Sc., copied in *Cosmos* for March, page 358, writes on this same line, verifying my experiments and conclusions Now, if you use porcelain inlays in place of gold, where you *must* use amalgam, your amalgam, if properly placed, will surprise you by its lasting qualities as well as bright color.

### Discussion.

Dr. S. D. Rambo: You speak about gold and amalgam not being tolerated in the same mouth. For years I have been placing amalgam under gold filling and I have found it a success, and I cannot let that paper pass without mentioning this fact.

Gold upon a layer of amalgam makes a successful filling. Of course, we have slight electric shocks, but they are not many. I cannot confine myself to the use of one metal for fillings.

Dr. B. H. Teague: Dr. Baum, of New York, was for many years an advocate of the theory that the cause of decay in teeth was electrolysis, but he was combatted by some of the most prominent men in the profession everywhere. In those cases where you suppose electrolysis causes decay of teeth you will find on close examination that the decay comes from some other cause.

